# Free-range versus conventional: A comparison of microbial composition and *Campylobacter* contamination in broiler carcasses after chilling

**DOI:** 10.1016/j.psj.2025.106111

**Published:** 2025-11-14

**Authors:** Sophie Hautefeuille, Sandrine Guillou, Agnès Bouju-Albert, Boris Misery, Béatrice Laroche, Nabila Haddad, Raouf Tareb

**Affiliations:** a Oniris, INRAE, SECALIM, 44300 Nantes, France; b Université Paris-Saclay, INRAE, MaIAGE, Jouy-en-Josas, France

**Keywords:** Meat microbiota, *Campylobacter*, Food safety, Broiler rearing, Slaughterhouse

## Abstract

Farming systems are known to significantly influence gut microbiota composition and *Campylobacter* colonization in live broilers. However, little is known about how these effects persist or change after slaughter, particularly in shaping the carcass microbiota. This study investigated the occurrence and enumeration of *Campylobacter*, the overall bacterial community structure in carcasses from conventional and free-range broilers, and the influence of slaughter batch timing (early *versus* late) on contamination and microbiota composition. A total of 314 post-chilling carcass samples were analyzed: 160 from conventional and 154 from free-range production systems, each obtained from two slaughterhouses specialized in their respective production methods. On each sampling day, half of the carcasses were collected from the first slaughtered batches, and the other half from the last. *Campylobacter* occurrence was higher in free-range carcasses (96.2 %) than in conventional ones (75 %), although mean counts were greater in conventional carcasses (1.8 log₁₀ CFU/ml) compared with free-range carcasses (1.3 log₁₀ CFU/ml). The timing of slaughter batches did not significantly affect *Campylobacter* contamination levels. Sparse Partial Least Squares-Discriminant Analysis (sPLS-DA) revealed a clear separation between the carcass microbiota of conventional and free-range broilers. Conventional carcasses were enriched in common gut- and processing-related genera, including *Aliarcobacter, Lactococcus*, and *Pseudarthrobacter*. Free-range carcasses displayed higher abundances of gut-associated taxa, including *Lysinibacillus, Comamonas*, and *Rikenellaceae* RC9 gut group, as well as genera not typically associated with the broiler gut or processing environments (*Ureibacillus, Pedobacter, Pseudoxanthomonas*). Within conventional systems, early batches were dominated by bacteria typical of processing environments (*Aliarcobacter, Chryseobacterium, Flavobacterium*). In contrast, later batches showed increased abundances of gut-associated and spoilage genera (*Clostridium, Bacillus, Romboutsia, Moraxella*). In free-range carcasses, later batches showed a higher abundance of the thermotolerant and biofilm-forming *Anoxybacillus*. Overall, these findings highlight distinct microbial signatures and *Campylobacter* contamination patterns between production systems and emphasize that slaughter-related factors, particularly processing time, further shape carcass microbiota composition.

## Introduction

As a cost‑effective and quickly producible protein source, poultry meat is projected to account for 41 % of global protein consumption by 2030, reflecting a 17.8 % growth over its present market share (OECD/FAO, 2021). Trends in broiler meat consumption have changed in recent years driven by the increasing consumer preference for foods produced through environmentally sustainable and animal welfare–conscious practices, although this trend may vary across regions and market segments (Amato et al., 2022; Campbell et al., 2025). Free-range and organic chicken meat have grown in popularity, with perceived improvements in animal welfare and husbandry. In France, free-range poultry accounted for approximately 14 % of broiler production in 2024 (with 13 % certified as Label Rouge and 1 % as organic), while over 75 % originated from intensively farmed poultry raised in confinement (conventional systems) (ANVOL, 2024). These alternative systems differ from conventional farming in several key aspects, including outdoor access, lower stocking density, older slaughter age, and stricter control over feed and medication use (Safitri and van Asselt, 2024).

Free-range systems are appreciated for their positive impact on animal welfare and environmental sustainability; however, they can encounter significant challenges concerning microbial safety (Campbell et al., 2025). The outdoor environment and increased exposure to wildlife complicate disease control and may facilitate the introduction of pathogens (McMullin, 2022). One notable pathogen is *Campylobacter* spp. responsible for campylobacteriosis. Campylobacteriosis is associated with consumption of broiler meat and has been the leading reported foodborne gastroenteritis in the European Union (EU) since 2005 (EFSA and ECDC, 2023). Several studies indicate that free-range broilers are more frequently colonized by *Campylobacter* due to their increased environmental exposure (Heuer et al., 2001; Hue et al., 2010; Näther et al., 2009). For example, Hue et al. (2010) reported a 100 % prevalence in caecal content and carcasses from Label Rouge and organic chickens in France, compared to 69.7 % and 84.7 %, respectively, in conventional broilers. Similarly, Rosenquist et al., 2013 found a higher prevalence in organic compared to conventional broiler carcasses in Denmark (54 % vs. 19.7 %). Nevertheless, other studies have found no significant differences between rearing systems (Economou et al., 2015; Iannetti et al., 2020), indicating that the relationship remains debated.

Research on the microbial ecology of broilers has increasingly focused on how farming systems influence gut microbiota. It is well-established that rearing conditions can affect the composition and diversity of intestinal microbiota (Ricke and Rothrock, 2020). For example, Varriale et al., 2022 found that broilers with outdoor access had higher species richness and diversity in their cecal microbiota. Similarly, Muyyarikkandy et al., 2023 reported that poultry from free-range systems had greater abundances of *Bacillota, Campylobacterota*, and *Bacteroidota* in their feces, while *Actinomycetota* were more prevalent in poultry raised in intensive farming systems. Most studies to date have been conducted at the farm level, focusing on live animals and fecal, or cecal samples. The impact of farming systems on carcass-level microbiota is still underexplored. A recent study by Punchihewage Don et al., 2023 is one of the few to compare carcass microbiota between conventional and organic systems. They found no significant differences in bacterial diversity, richness, or dominant phyla between the two groups. Processing steps during slaughter, particularly scalding, plucking, and evisceration, can significantly alter the microbial profile of carcasses (Althaus et al., 2017; Rosenquist et al., 2006). These steps can not only increase contamination levels on carcasses but may also obscure initial microbial differences associated with various rearing systems. Additionally, the timing of slaughter—whether early or late batches—can affect contamination levels due to cross-contamination along the slaughter line, especially with shared equipment like scalding tanks and evisceration tools. For example, Wang et al., 2019 reported a gradual increase in the number of mesophilic aerobic bacteria on carcasses throughout the day. However, other studies have found no significant effect of slaughter time on *Campylobacter* or total viable count (TVC) levels (Zweifel et al., 2015), suggesting that this factor also requires further clarification.

In this study, we aim to investigate *Campylobacter* contamination and microbial diversity on broiler carcasses, considering the farming systems and the timing of slaughter batches. Understanding these factors is essential for enhancing the safety and shelf-life of broiler products. To address this, the present study aimed to (i) compare *Campylobacter* contamination levels and bacterial composition on carcasses from conventional and free-range (Label Rouge) broilers, and (ii) assess the influence of slaughter batch timing on these microbial parameters.

By examining these variables at the carcass level, this study provides valuable insights into how both rearing and processing conditions affect contamination and microbial ecology in broiler meat.

## MATERIALS AND METHODS

The data generation methods used in this study are detailed in the published data paper (Hautefeuille et al., 2024) https://doi.org/10.1016/j.dib.2024.110858. The generation method described for conventional carcasses is identical to that used for free-range carcasses.

### Broiler carcass collection

Between September 2022 and January 2023, 480 conventional broiler carcasses (chicken age: 35) were collected after chilling over ten non-consecutive slaughter days in the same slaughterhouse. One year apart, from September 2023 to February 2024, 477 Label Rouge (free-range) carcasses (chicken age: 85) were similarly collected after chilling, also over ten non-consecutive slaughter days but in another slaughterhouse because, in France, slaughterhouses are mostly specialized in slaughtering broilers raised according to a single type of rearing system.

For each farming system and on each of the ten days of slaughter, 48 carcasses were sampled: 24 from the first batch of the day (between 02:15-07:15 and 00:15-10:53 am for the conventional and Label Rouge carcasses, respectively) and 24 from subsequent batches processed later in the day (between 8:55-12:40 and 03:00-12:51 am for the conventional and Label Rouge carcasses, respectively). These time ranges represent the overall variability observed across the ten sampling days, as slaughter timing depended on slaughterhouse own logistics and the number of flocks processed each day. The time, date, and processing speed (slaughter rate) of each slaughter batch are provided in Supplementary Table 1.

### Sample preparation for microbiological analysis and bacterial community sequencing

Half of each carcass was sampled and kept overnight at 4°C before analysis. Each half-carcass was then rinsed in 700 ml of buffered peptone water in a Stomacher bag, kneaded manually for about 15 seconds, and shaken at 150 rpm for five minutes in an incubator (Infors HT Minitron) at ambient temperature. The rinses from three different half-carcasses were pooled (making a sample) in a sterile 2 L beaker containing a sterile magnetic stir bar and mixed on a magnetic stirrer plate for 15 seconds to ensure a uniform sample.

DNA extractions, as well as quantifications of *Campylobacter* and total viable count (TVC), were performed on a total of 319 pooled rinse samples: 160 from conventional broiler carcasses and 159 from free-range broiler carcasses. Each sample represented a pooled rinse of three half-carcasses as described below, totaling 480 conventional half-carcasses and 477 free-range half-carcasses. All analyses were conducted on these pooled half-carcass samples.

#### Campylobacter quantification

*Campylobacter* quantification was performed using selective CASA (*Campylobacter* Selective Agar) agar (Biomérieux, France) (Ahmed et al., 2012; Andritsos et al., 2020; Le Bars et al., 2011) after serial tenfold dilutions in buffered peptone water (Laboratoire Humeau, France). Plates were incubated at 42°C for 48 hours using the jar gassing system Macs Mics (AES Chemunex, don whitley scientific), *i.e.*, in sealed metal jars flushed with a gas mixture of 5 % O₂, 10 % CO₂, and 85 % N₂. Bacterial concentrations were determined by counting dark red colonies. The quantification limit was 0.7 log_10_ CFU/ml, corresponding to fewer than 10 *Campylobacter* colonies per Petri dish. All data below this threshold were considered as censored data.

#### Total viable count quantification

Total Viable Count (TVC) was quantified to assess the bacterial load of both conventional and free-range carcasses. PCA plates (VWR, France) were inoculated with 100 µL of each serially diluted pooled rinse (from 10^−1^ to 10^−5^) and incubated at 30°C for 48 hours. Bacterial concentrations were calculated according to the NF EN ISO 7218 standard.

### Bacterial community sequencing

#### DNA extractions

For DNA extraction, bacterial pellets were obtained from 160 mL of rinses through sequential centrifugations: 600 g for 5 minutes to remove debris, followed by 10,000 g for 10 minutes to concentrate cells. The pellets were then resuspended in 1 mL of peptone water and centrifuged again (10,000 g, 5 minutes).

DNA was extracted using the PowerFood Microbial Kit (Qiagen, France) with slight modifications: pellets were resuspended in 450 µL of Solution MBL, agitated in PowerBead Tubes for 30 seconds, and centrifuged. The supernatants were mixed with IRS Solution, incubated on ice for 30 minutes, and centrifuged before being combined with MR Solution and loaded onto MB Spin Columns. After washing with PW Solution and ethanol, the membranes were dried at 50°C, and DNA was eluted in 100 µL of ultrapure water.

DNA concentration and purity were assessed using an Implen Nano Spectrophotometer® N50, and Tuf qPCR was performed on selected samples to check for amplification inhibitors.

For a dozen samples with low A260/A230 ratios and amplification inhibitors, an additional purification step was performed. Ethanol (250 µL) was added to the 100 µL extract and incubated on ice for 10 minutes, followed by two centrifugations (16,000 g, 30 min, 4°C). The pellet was then air-dried (30 min), resuspended in 30 µL of preheated water (50°C), and incubated for 5 minutes at 50°C before storage at 4°C. This step improved DNA quality by increasing the A260/A230 ratio and reducing inhibitors, ensuring more reliable PCR and qPCR amplifications.

#### 16 rRNA sequencing and raw sequence analysis

A total of 20 µL of each DNA sample was sent to Eurofins Genomics for 16S rRNA sequencing on an Illumina MiSeq, with conventional and free-range broiler samples sequenced one year apart. A mock community, serving as an *in situ* positive control and containing a mixture of several known bacterial species at defined concentrations, was used to assess sequencing accuracy and validate the bioinformatics workflow. The mock community included 3 × 10⁸ cells each of *Brochothrix thermosphacta, Carnobacterium maltaromaticum, Hafnia alvei, Lactobacillus sakei, Pseudomonas fluorescens, Serratia marcescens*, and 3 × 10⁶ cells of the reference strain *Campylobacter jejuni* 81-176. The V3-V4 regions were PCR-amplified, cleaned, and sequenced using 2 × 300 bp paired-end reads. Eurofins preprocessed the data by trimming adapters, applying quality filtering (Q30 > 75 %), and merging reads.

Bioinformatics analysis was performed on the Galaxy Migale platform using FROGS for denoising and clustering, with conventional and free-range samples processed together to avoid computational biases between datasets (Escudié et al., 2018). Taxonomic assignment was done via BLAST using the SILVA database (Quast et al., 2013). ASVs were filtered based on their presence in at least 25 % of the samples (80 out of 319 samples) to focus analysis on biologically meaningful or consistently observed ASVs and an abundance threshold of 0.0005. After testing several filters, this filtering approach preserved an adequate number of ASVs while avoiding the elimination of informative species or the retention of ASVs potentially associated with sequencing errors or noise. After analyzing the rarefaction curves, five free-range samples were excluded because their sequencing depth was insufficient to capture the expected diversity. As a result, 314 out of 319 samples were retained. The ASV table was rarefied on the online instance of Easy16S (Midoux et al., 2024) (https://shiny.migale.inrae.fr/app/easy16S).

### Unsupervised statistical analysis

#### Campylobacter and TVC levels in conventional or free-range carcasses

*Campylobacter* and TVC quantification data were expressed in CFU/ml of carcass rinse, including left-censored data below 5 CFU/ml. After log_10_ transformation (resulting in a quantification limit of 0.7 log_10_ CFU/ml), the *Campylobacter* and TVC levels of the conventional and free-range carcasses were fitted to LogNormal, Normal, Logistic, Weibull and Gamma distributions. The best-fitting models among Lognormal, Normal, Logistic, Weibull, and Gamma, were selected by using the fitdistrplus R package, according to the Akaike and Bayesian information criteria (AIC, BIC, Supplementary Table 2) (Delignette-Muller and Dutang, 2015) (1.2.2 version).

According to AIC, the Logistic model best described the distribution of *Campylobacter* contamination level in conventional (excluding censored data likely to include true zeros) and free-range carcasses (Supplementary Table 2). For TVC, Logistic distribution was also retained. Because *Campylobacter* data for conventional carcasses included both contaminated and non-contaminated samples, a mixed distribution model combining a Bernoulli component (presence/absence) and a censored logistic component was fitted to capture both features of the data. The adjustment of the mixture distribution was performed in one row by maximizing the likelihood of the data using the optim function.

Uncertainty in distribution parameters was assessed using a bootstrap resampling approach (1,001 replicates), and simulated distributions were generated using the mc2d R package to compute confidence intervals of bacterial loads. Finally, *Campylobacter* and TVC levels were compared between conventional and free-range carcasses using Wilcoxon rank-sum tests (rstatix R package) (Kassambara, 2023).

### *Campylobacter and TVC levels between slaughter batches in conventional and free-range carcasses*

The levels of *Campylobacter* and TVC between the first and later batches, both in conventional and free-range carcasses, were compared using non-parametric Wilcoxon tests (rstatix R package) (Kassambara, 2023).

#### Bacterial community composition and diversity between conventional and free-range carcasses

To estimate alpha diversity between conventional and free-range broiler carcasses, Shannon, InvSimpson, Observed and Chao1 diversity indices were calculated using R phyloseq package (McMurdie and Holmes, 2013) and compared with ANOVA test (stats R package) (Team, 2014). To test whether beta diversity of the bacterial community was different between conventional and free-range carcasses, we conducted Principal Coordinates Analysis (PCoA) and Permutational multivariate analysis of variance (PERMANOVA, 999 permutations) on the Bray-Curtis distance dissimilarity matrices using adonis2 function in the vegan package (Oksanen et al., 2013). PERMANOVA assumes homogeneity of groups dispersions (variance around the centroid) (Anderson et al., 2006). We used the Betadisper function to test whether this assumption was met (vegan package).

Sunburst diagrams were generated using Python (v6.0.0) and the plotly.express library (Plotly Technologies Inc., Montréal, Canada, https://plotly.com/python/plotly-express/) to illustrate the taxonomic distribution based on the percentage of abundances (and relative abundance) in both conventional and free-range broiler carcass samples. A predefined color palette was assigned to different phyla to ensure visual consistency. Each chart was exported in HTML format for interactive exploration and available through this doi: https://doi.org/10.57745/VTJBSX.

### Supervised statistical analysis: discriminating microbiota according to rearing methods and slaughter time

To discriminate the microbiota of broiler carcasses based on the rearing method and slaughter time, we performed two sPLS-DA discriminant analysis using Rstudio (2023.03.1 + 446 version) (Team, 2014). One analysis aimed to distinguish the bacterial composition between conventional *versus* free-range carcasses, and the second one, first slaughter batches (beginning of the day) from later batches from both conventional and free-range broiler carcasses.

To do so, abundance tables were transformed using centered-log ratio (CLR) transformed with an offset of one, applying the logratio.transfo function. The sPLS-DA function was then used to identify the most discriminant ASV by constructing a one-component model and selecting the optimal number of variables to retain via the tune.splsda function. For each analysis, the model was tuned using 10-fold cross-validation repeated 500 times to determine the optimal number of components and ASVs. The average balanced classification error rate served as the selection criterion. Finally, the model’s performance was assessed based on its AUC, evaluated using the perf function with 10-fold cross-validation repeated 500 times.

## Results

### Campylobacter and TVC levels in conventional and free-range broiler carcasses

**Fig. 1:**
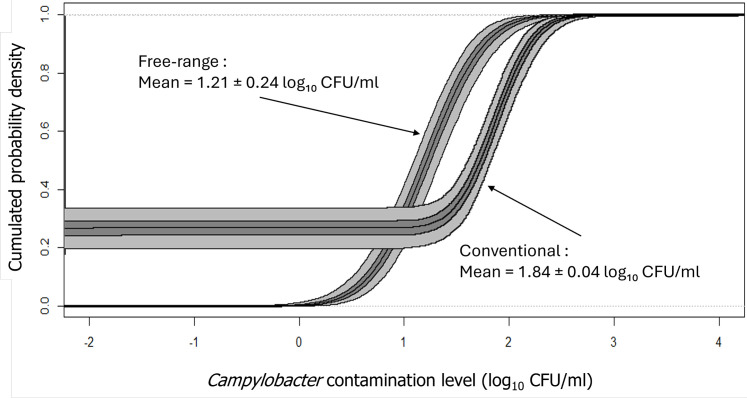
Distribution of *Campylobacter* contamination levels on conventional and free-range carcasses (log_10_ CFU/ml of carcass rinse). The figure shows the Logistic distribution of *Campylobacter* contamination levels on conventional carcasses and on free-range carcasses. Light areas represent the variability range, while darker areas indicate uncertainty intervals.

**Fig. 2:**
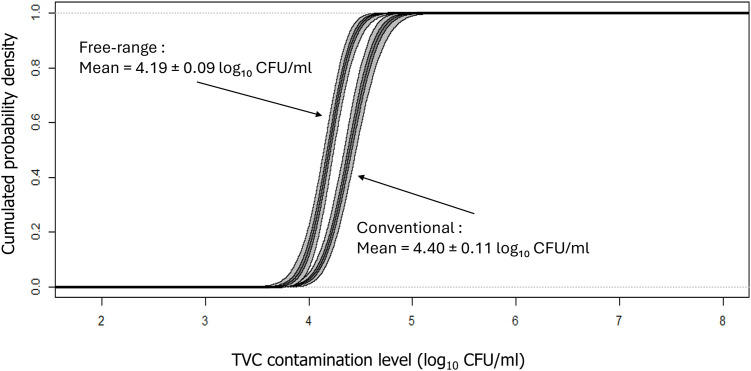
Distribution of Total Viable Count (TVC) levels on conventional and free-range carcasses (log_10_ CFU/ml of carcass rinse). The figure shows the Logistic distribution of TVC levels on conventional carcasses and on free-range carcasses. Light areas represent the variability range, while darker areas indicate uncertainty intervals.

Our results indicated that *Campylobacter* was present in 75 % of conventional broiler carcasses and 96.2 % of free-range broiler carcasses, showing a higher occurrence in the free-range group. The contamination levels of conventional broiler carcasses averaged 1.8 ± 0.04 log₁₀ CFU/ml of carcass rinse, while the levels for free-range carcasses averaged 1.2 ± 0.4 log₁₀ CFU/ml (Fig. 1). The difference of the levels of *Campylobacter* was significant (P < 0.05). In terms of Total Viable Counts (TVC), conventional carcasses had an average of 4.4 ± 0.2 log₁₀ CFU/ml of carcass rinse, compared to 4.2 ± 0.2 log₁₀ CFU/ml for free-range carcasses (Fig. 2). The TVC levels were significantly higher in conventional carcasses (P < 0.001).

### Bacterial diversity and composition between conventional and free-range broiler carcasses

#### Bacterial diversity

**Fig. 3:**
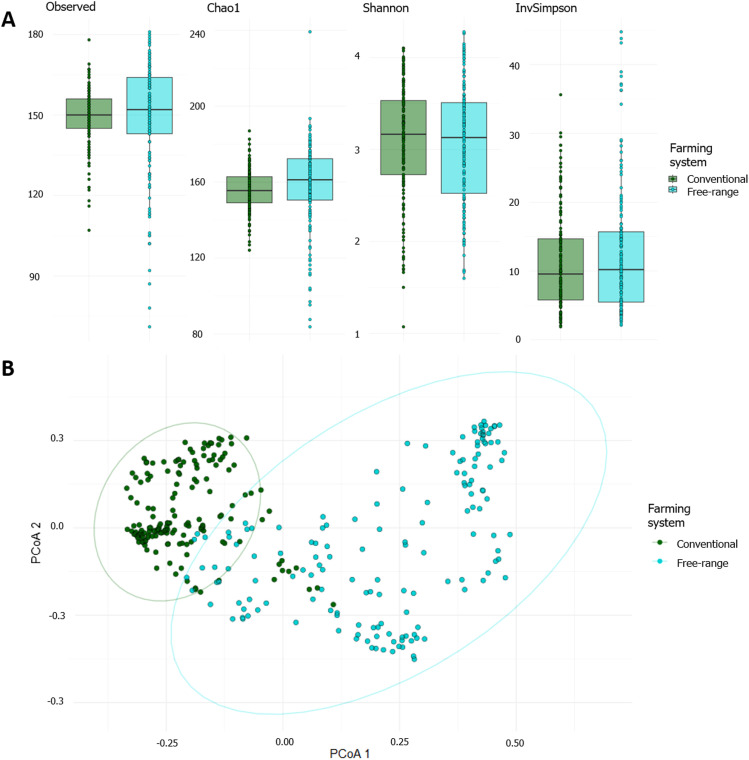
Alpha and beta-diversity between conventional and free-range carcasses. **(A)** Alpha-diversity (observed, chao1, Shannon and InvSimpson) metrics between conventional (green) and free-range (turquoise) carcasses. **(B)** Beta-diversity (Bray-Curtis dissimilarity) between conventional (green) and free-range (turquoise) carcasses. Ellipses are shown for each farming system with the corresponding colors.

Species richness, measured by Observed and Chao1 indices, and bacterial diversity, measured by Shannon and InvSimpson were not different between the conventional and free-range carcasses (ANOVA, P = 0.055, P = 2.21, P = 0.19 and P = 2.23, respectively) (Fig. 3A). However, beta-diversity (Bray-Curtis dissimilarity) differed (PERMANOVA, P < 0.001), by showing greater beta-diversity in free-range carcass samples (Fig. 3B). However, the Betadisper test, assessing dispersion differences between conventional and free-range samples, was significant (P < 0.05). This suggests that the PERMANOVA results may be influenced by the heterogeneity in the sample distribution between conventional and free-range groups.

#### Bacterial composition

**Fig. 4:**
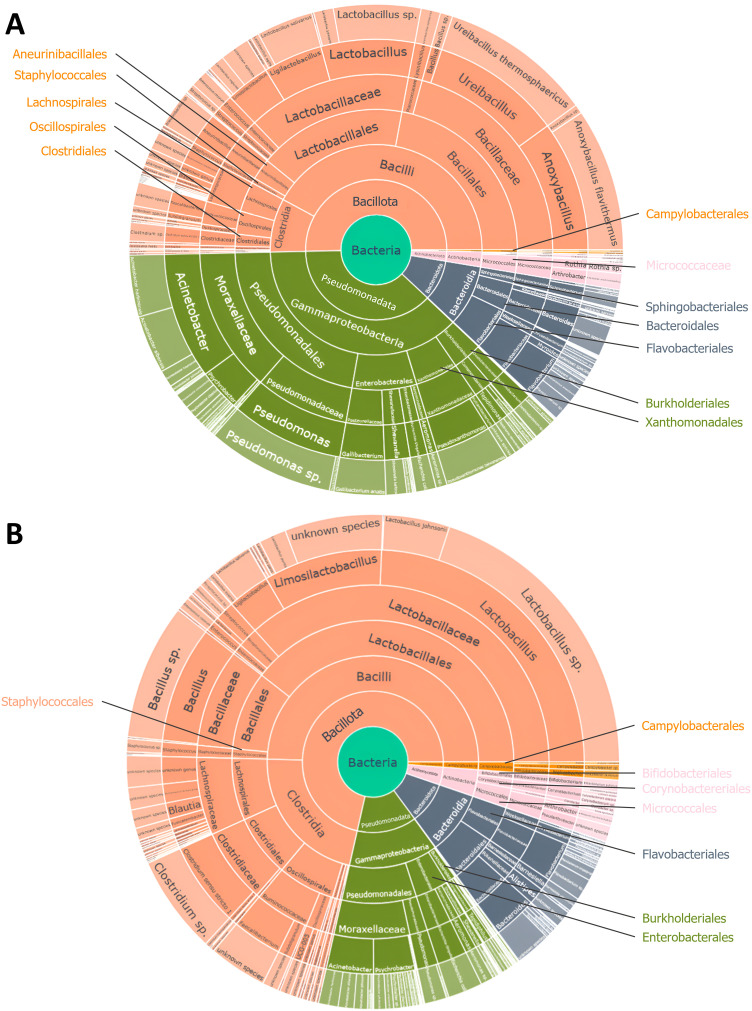
Bacterial composition by taxonomic rank of free-range **(A)** and conventional **(B)** broiler carcasses. The different layers of these pie charts represent the abundance (in percentage) of all the taxa at each taxonomic rank, starting from the center (kingdom) and moving outward to the species at the outermost layer. This figure is interactive and can be found in https://doi.org/10.57745/VTJBSX.

**Fig. 5:**
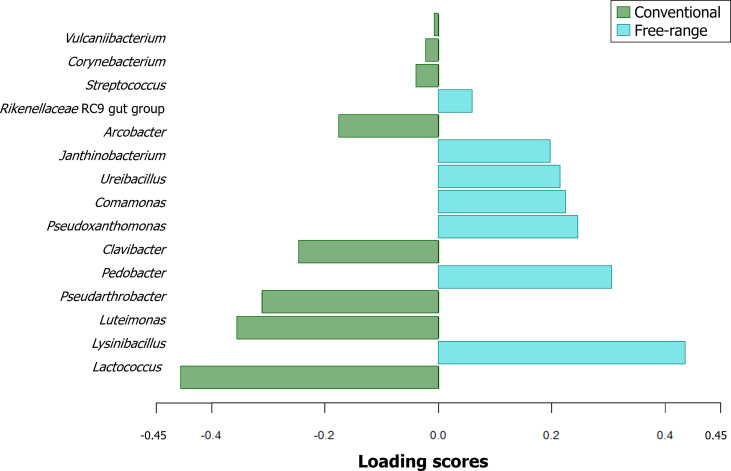
**Discriminant analysis (sPLS-DA) of the bacterial genera between conventional and free-range carcasses.** The loading scores (positive and negative) indicate group assignment (*i.e.*, conventional *vs*. free-range). This analysis highlights differences in bacterial genera composition between conventional and free-range broiler carcasses.

The analysis of the bacterial composition of broiler carcasses from conventional and free-range farming systems revealed 5 phyla, 7 classes, 18 orders, 43 families, 79 genera, and 136 species (Fig. 4A and B). The five phyla identified were *Bacillota, Pseudomonadota, Bacteroidota, Campylobacterota*, and *Actinomycetota. Bacillota* and *Pseudomonadota* were the dominant phyla in both systems, though they appeared in different proportions. Specifically, *Bacillota* predominated in conventional carcasses (71.4 %) compared to 50.3 % in free-range carcasses, while *Pseudomonadota* were more abundant in free-range samples (37.5 % *versus* 13.6 %). To further differentiate the bacterial communities between the two production systems, a sparse Partial Least Squares Discriminant Analysis (sPLS-DA) was conducted. The selected model consisted of one component and 15 genera, exhibiting a strong robustness with an Area Under the Curve (AUC) of 1.0. The top 15 discriminant genera contributing to the first component are presented in Fig. 5. Carcasses from conventional farming are characterized by a higher abundance of eight bacterial genera, mainly including those from the phylum *Bacillota* (*Lactococcus, Streptococcus*, and *Corynebacterium*), the phylum *Pseudomonadota* (*Luteimona*s and *Vulcaniibacterium*), the phylum *Actinomycetota* (*Pseudarthrobacter* and *Clavibacter*), and the phylum *Campylobacterota* (*Aliarcobacter*, previously *Arcobacter*). In contrast, carcasses from free-range farming systems exhibited a dominance of bacterial genera belonging to the phylum *Pseudomonadota* (*Pseudoxanthomonas, Comamona*s, and *Janthinobacterium*), the phylum *Bacillota* (*Lysinibacillus* and *Ureibacillus*), and the phylum *Bacteroidota* (*Pedobacter* and the *Rikenellaceae* RC9 gut group). Finally, although present at low abundance, *Campylobacter* spp. was detected, using 16S RNA metabarcoding, in both conventional and free-range carcasses (0.42 % and 0.13 %, respectively). Taxonomic analysis revealed that *Campylobacter* sequences were multi-affiliated with *C. jejuni, C. coli*, and *C. upsaliensis*.

### Influence of slaughter time on *Campylobacter*, TVC levels and microbial diversity

The *Campylobacter* levels between the first slaughter batches and later batches were not different (Wilcoxon test, P > 0.5). However, TVC levels (∼4 log CFU/mL) were slightly influenced by slaughter time in free-range carcasses (Wilcoxon test, P = 0.003) but not in conventional ones (Wilcoxon test, P = 0.09). Species richness, measured by Observed and Chao1 metrics, was higher in later slaughter batches in free-range carcasses (ANOVA, P = 0.02 and 0.01, respectively) (Supplementary Figure 1A). Bacterial diversity (Shannon and InvSimpson) was not different among batches (data not shown). For conventional carcasses, alpha diversity by Observed metrics was higher among later batches, but diversity by InvSimpson was lower in later batches (ANOVA, P = 0.04 and 0.01, respectively) (Supplementary Figure 1B). Chao1 and Shannon metrics were not different among batches (data not shown). Beta diversity was different among first and later batches in both conventional and free-range samples (PERMANOVA, P = 0.001) (Supplementary Figure 1C and 1D).

### Discriminating microbiota associated with slaughter time in conventional and free-range broiler carcasses

**Fig. 6:**
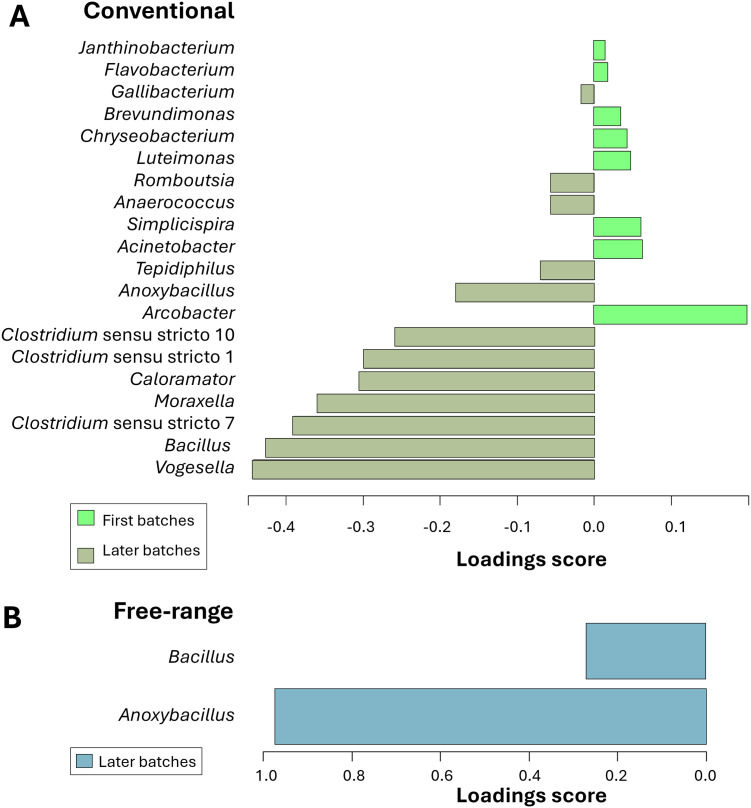
Discriminant analysis (sPLS-DA) of microbiota based on slaughter time in conventional and free-range carcasses. **(A)** Differentiation of microbiota from conventional broiler carcasses collected in the first slaughter batches (light green) versus those from later batches (kaki). **(B)** Differentiation of microbiota from free-range broiler carcasses collected in the first slaughter batches versus those from later batches (grey blue). The loading scores (positive and negative) indicate group assignment (*i.e.*, first batches *vs*. later batches). This analysis highlights differences in bacterial composition between first and late slaughter batches in both conventional and free-range broiler carcasses.

We performed discriminant analysis (sPLS-DA) to identify bacterial genera that most effectively differentiated the first and the later slaughter batches in conventional and free-range broiler carcasses. For the conventional carcasses, the selected model, comprising one component and 50 genera, demonstrated strong robustness with an AUC of 0.881 ± 0.003. For reason of figure clarity, the top 20 discriminant genes contributing to the first component were plotted (Figure 6). The other discriminant genera are listed in the Supplementary Table 3. The first slaughter batches were characterized by a dominance of several genera, such as, *Aliarcobacter* (previously *Arcobacter*), *Acinetobacter, Simplicispira, Luteimonas, Chryseobacterium, Brevundimonas* and *Flavobacterium* (Figure 6A). In contrast, later batches were characterized by a dominance of different genera, including *Vogesella, Bacillus, Clostridium, Moraxella, Anoxybacillu*s, *Tepidiphilus, Anaerococcus* and *Romboutsia* (Figure 6A).

For the free-range carcasses, the selected model also demonstrated robustness, with an AUC of 0.779 ± 0.002. This model included one component and two genera. The two genera that most strongly discriminated the later batches from the first batches were *Anoxybacillus* and *Bacillus* (Figure 6B).

## DISCUSSION

Several studies have shown that farming systems can influence *Campylobacter* colonization and the microbiota composition of broiler gut (Hue et al., 2010; Varriale et al., 2022). However, the *Campylobacter* contamination and microbiota composition of the carcass in relation to the farming system has been less studied. Comparing carcass microbiota can provide insights into the persistence of farming-related microbial signatures after processing and their potential impact on meat quality, spoilage, and food safety.

In this study, we first assessed *Campylobacter* contamination levels and the microbial composition and diversity in conventional and free-range (Label Rouge) carcasses. Comparing microbiota of conventional and free-range carcasses allowed us to assess to what extent operational factors (farming and slaughter systems) can influence both their contamination levels and microbiota composition. Secondly, we evaluated the effect of slaughter time on these contamination levels and bacterial composition. This second objective was to assess whether *Campylobacter* and TVC levels were higher among later batches, potentially accompanied by greater diversity and species richness as the slaughter day progressed, along with a distinct microbial signature compared to the first batches.

### *Campylobacter* occurrence and contamination levels between broiler types

Our results showed that *Campylobacter* occurrence (samples above 0 CFU/ml) was higher among free-range carcasses, but the contamination levels were higher in conventional carcasses. Similarly to our results, lower *Campylobacter* spp. levels have already been reported in cecal and neck skin samples of broilers carcasses at slaughterhouse, from free-range farms compared to conventional (2.42 *vs.* 3.15 log_10_ CFU/g) (Iannetti et al., 2020). In general, free-range farming systems are associated with higher prevalence in poultry, without necessarily having higher contamination levels (Heuer et al., 2001; Hue et al., 2010; Näther et al., 2009). This is mainly explained by the increased exposure of poultry to environmental sources of *Campylobacter* (water, soil, and other birds) and other livestock or wild fauna in the vicinity of the flocks. However, most of the studies compare the cecal or fecal *Campylobacter* levels between the different farming systems. In contrast, our study focused on carcass contamination, which is also influenced by slaughtering processes specific to each facility. Moreover, it is important to note that in our study, the slaughterhouse from which the conventional broiler samples were collected had a history of frequent non-compliant self-monitoring results (neck samples exceeding 1,000 CFU/g of *Campylobacter* spp.). As parallel sampling of caeca - which reliably reflects colonization rates at the farm level - was not performed the influence of the different farming systems can be inferred but not conclusively confirmed.

### Microbiota composition and diversity between broiler types

Microbial composition of carcasses and total viable counts levels also differed between the conventional and free-range carcasses in this study. This study highlights differences between conventional and free-range broiler carcasses in terms of microbial contamination and bacterial community composition. Taxonomic analysis revealed distinct microbial signatures associated with each production system. The sparse Partial Least Squares Discriminant Analysis (sPLS-DA) identified 15 genera discriminating between systems. The conventional carcasses exhibited higher abundances of three genera from the phylum *Bacillota: Lactococcus, Streptococcus*, and *Corynebacterium*. These genera are common constituents of the broiler gut microbiota and are also present in litter and the airborne microflora of broiler houses. Furthermore, they have been detected in the air of poultry slaughterhouses, specifically in areas such as reception and chilling (Ellerbroek, 1997; Koort et al., 2006; Lauritsen et al., 2019; Song et al., 2022; Vihavainen et al., 2007; Wang et al., 2020; Xiao et al., 2017). Importantly, *Aliarcobacter* (previously *Arcobacter*) was also among the more abundant genera in conventional carcasses. This genus, particularly *A. butzleri*, represents a major food safety concern. It is widespread in broiler production and poultry meat, having been isolated from various sources, including feces, water, and slaughterhouse environments. Its persistence on processing equipment, high prevalence in broiler intestines (up to 62% in colonic contents), and ability to survive cleaning and chilling make it a significant risk factor for cross-contamination (Botta et al., 2024; Mateus et al., 2021; Niyayesh et al., 2024; Schönknecht et al., 2020). On the other hand, exclusively environmental genera such as *Luteimonas* and *Pseudarthrobacter* were also more abundant in the conventional carcasses' microbiome. The presence of *Pseudarthrobacter* aligns with reports showing that air chilling can introduce or favor the persistence of environmental bacterial taxa on broiler carcasses. This genus, primarily associated with soil and water, has been detected on chicken carcasses—particularly after air chilling in commercial processing lines—where its abundance tends to increase. These findings indicate that the source of *Pseudarthrobacter* is primarily the processing environment (*e.g*., contact surfaces and equipment) and not the chicken gut (Chen et al., 2020).

In contrast, free-range carcasses were enriched in genera typically associated with the broiler gut microbiome, such as *Lysinibacillus, Comamonas, and the Rikenellaceae* RC9 gut group, as well as processing environment genera such as *Janthinobacterium*. The detection of the *Rikenellaceae* RC9 gut group and *Lysinibacillus* is consistent with previous reports describing them as common residents of the chicken cecal and ileal microbiota, respectively, and as taxa frequently detected in fresh fecal samples of broiler chickens (Chen et al., 2020; Choi and Kim, 2023; Huang et al., 2018; Qiu et al., 2022; Viso et al., 2021). *Comamonas* is commonly detected within feather follicles on chicken skin after slaughter and chilling. Critical processing stages such as evisceration, defeathering, and chilling facilitate its transfer from gastrointestinal and environmental sources to carcass surfaces (Zhang et al., 2022)*. Janthinobacterium* is frequently found on processing surfaces and in floor drains, where it often co-occurs with *Pseudomonas and Acinetobacter* (Fagerlund et al., 2025, 2021)*.* Based on current knowledge, *Ureibacillus, Pedobacter*, and *Pseudoxanthomonas* genera, identified as discriminants for free-range carcasses, are not considered ecologically abundant or relevant in the broiler chicken gut, on poultry meat, or during the slaughter process. Instead, they are commonly reported in other environments, such as soil, compost, and water (including drinking water) (Bansal et al., 2022; Simoes Junior and MacLea, 2021; Viana et al., 2018).

Overall, these findings demonstrate how both rearing and processing environments influence the microbiota present in chicken carcasses. In our study, chickens from each farming system (either conventional or free-range) were slaughtered at separate facilities, with all broilers of the same type, processed in the same slaughterhouse. Determining whether the differences in microbiota composition between conventional and free-range broilers originate from the initial gut microbiota associated with each farming system or from the specific slaughtering practices of each facility remains a complex challenge. Slaughter protocols may vary across the facilities, which may help explain the differences in the composition of the observed carcass microbiota, not associated with the rearing system. This variation is particularly evident during evisceration. If the machinery is not set up correctly, it can cause increased ruptures in intestinal contents, leading to higher levels of contamination of the carcasses with gut microbiota, including *Campylobacter*. Another important observation is that the slaughterhouse specializing in conventional broiler processing is significantly older than the facility dedicated to free-range broilers. As a result, the newer slaughterhouse for free-range poultry may have benefited from better-controlled slaughtering and cleaning processes. For example, a comparative study of microbiota from chiller tanks and carcasses across three slaughterhouses revealed a significantly higher abundance of *Pseudomonadota* in one establishment compared to the others (Kunert-Filho et al., 2022), underscoring the substantial impact of the slaughterhouse environment. Additional confounding factors related to the production system, such as slaughter age and bird size, may also contribute to the variations observed. For instance, the slaughter age differs significantly between conventional and Label Rouge broilers, with approximately 35 days for conventional birds compared to a minimum of 81 days for Label Rouge. Previous studies have shown that broiler age can influence the composition of the gut microbiota (Li et al., 2022; Marcato et al., 2024).

### Impact of slaughter time (batches) on *Campylobacter* levels and microbiota

Studies agree that while the time of day may not significantly influence contamination on its own, factors like cross-contamination during processing and equipment hygiene may contribute to variability in microbial contamination between slaughter batches (Wang et al., 2019; Zweifel et al., 2015). Throughout the day, the slaughterhouse can accumulate organic matters and bacteria from previous batches, despite intermittent cleaning procedures (Marmion et al., 2023; Perez-Arnedo and Gonzalez-Fandos, 2019; Reiche et al., 2025). *Campylobacter* can survive and spread through the chilling water, conveyors, and processing equipment (Rasschaert et al., 2020; Seliwiorstow et al., 2016, 2015). For example, several studies have highlighted the risk of *Campylobacter* transmission to a subsequent negative batch during slaughter. When the preceding positive batch had high contamination levels, *Campylobacter* was detected on carcasses from the following negative batch, although at significantly lower concentrations (Seliwiorstow et al., 2016, 2015). In our study, the *Campylobacter* contamination levels were similar between the first and later batches for both conventional and free-range carcasses. Consistent with the study of Zweifel et al., 2015, we did not observe any differences in *Campylobacter* levels according to slaughter time, for both conventional and free-range carcasses.

However, the observed species richness was higher in the later batches for both conventional and free-range carcasses microbiota. In contrast, diversity, measured by Shannon index, did not differ between batches. It can be hypothesized that species richness increases as the day progresses during slaughter, likely due to the accumulation of bacteria on the processing line and in the scalding water, without altering the diversity and species distribution.

Additionally, the discriminant analysis successfully differentiated microbiota based on slaughter time. The early batches of conventional carcasses were characterized by higher abundances of *Aliarcobacter* (previously *Arcobacter*), *Chryseobacterium, Flavobacterium, Acinetobacter*, and *Simplicispira*, belonging to the phyla *Pseudomonadota, Campylobacterota*, and *Bacteroidota*. These taxa are typically associated with environmental sources or, in the case of *Campylobacterota*, with the broiler gut (Botta et al., 2024; Wages et al., 2019; Zhang et al., 2020). Conversely, later batches showed higher abundances of *Clostridium, Bacillus, Romboutsia*, and *Anaerococcus*, taxa mainly linked to the gut microbiota (Awasthi et al., 2018; Lin et al., 2017; Zhang et al., 2021; Zhu et al., 2022). They also exhibited higher abundances of *Vogesella*, a common aquatic Gram-negative genus (Yu et al., 2020), and *Moraxella*, a frequent spoilage organism in meat processing environments, often transferred from equipment or personnel to carcass surfaces (Zwirzitz et al., 2020).

In free-range carcasses, later batches were dominated by ASVs affiliated with *Anoxybacillus* and *Bacillus*. Notably, *Anoxybacillus* is thermotolerant (optimal growth at 50–65 °C), capable of surviving heat treatments, and forming biofilms during processing, which may explain its persistence on meat after scalding and singeing (Zwirzitz et al., 2020).

These findings suggest that the first slaughter batches were primarily more enriched by environmental bacteria, while later batches exhibit a shift toward bacteria of digestive origin. This pattern could be explained by the gradual accumulation of gut bacteria along the slaughter line, facilitating the spread of these bacteria as the day progresses. First slaughter batches, processed earlier in the day, are likely less exposed to digestive-origin bacteria, as the accumulation of these bacteria on surfaces (such as handling equipment, and washing water) is still limited. However, to confirm this, data on water quality and bacterial load on surface contact through the processing line at different times of the day would be needed.

## Conclusion

Our study revealed significant differences in *Campylobacter* contamination and microbiota composition between conventional and free-range carcasses. These differences show that carcasses from different farming contexts exhibit distinct microbiotic signatures and *Campylobacter* contamination at the end of slaughter. Our results also indicate that while carcass contamination varies across farming practices, slaughter processes—such as slaughter time—can also modulate the carcass microbiota at the end of the food chain. In France, the specialization of slaughterhouses according to the farming system is a structural constraint that we had to account for in our study. Nevertheless, this specialization highlights the importance of considering slaughterhouse-related effects when interpreting differences between farming systems and offers an opportunity for future studies to better disentangle farm- and processing-related influences on carcass microbiota and contamination.

## Funding

This work was supported by the ANR ESCAPE Project, Grant ANR-21-CE21-0008, operated by the French Agence Nationale de la Recherche. SH was granted for PhD study through this agency and this project.

## Data availability

The datasets analyzed during the current study are available in the INRAE institutional data repository. Raw sequencing data for conventional carcass samples are available at https://doi.org/10.57745/WUII9H, and at https://doi.org/10.57745/OGBIIU for free-range carcass samples. ASV and ASV multi-affiliation tables (rarefied and non-rarefied), taxonomy tables, metadata, bacterial quantification and interactive figures are available at https://doi.org/10.57745/VTJBSX.

## CRediT authorship contribution statement

**Sophie Hautefeuille:** Writing – original draft, Visualization, Validation, Project administration, Methodology, Investigation, Formal analysis, Conceptualization. **Sandrine Guillou:** Writing – review & editing, Validation, Supervision, Project administration, Methodology, Investigation, Formal analysis, Conceptualization. **Agnès Bouju-Albert:** Writing – review & editing, Supervision, Investigation. **Boris Misery:** Writing – review & editing, Supervision, Investigation. **Béatrice Laroche:** Writing – review & editing, Validation, Supervision, Resources, Methodology, Conceptualization. **Nabila Haddad:** Writing – review & editing, Validation, Supervision, Resources, Project administration, Methodology, Investigation, Funding acquisition, Conceptualization. **Raouf Tareb:** Writing – review & editing, Validation, Supervision, Project administration, Methodology, Investigation, Conceptualization.

## Disclosures

The authors declare that they have no known competing financial interests or personal relationships that could have appeared to influence the work reported in this paper.

## Appendix Supplementary materials

Image, application 1

## Footnotes

Supplementary material associated with this article can be found, in the online version, at doi:10.1016/j.psj.2025.106111.
