# Supplementary material for: Free-range versus conventional: A comparison of microbial composition and Campylobacter contamination in broiler carcasses after chilling
Source: Poult Sci. 2025 Nov 14;105(1):106111. doi: 10.1016/j.psj.2025.106111 (PMC12723032; doi:10.1016/j.psj.2025.106111)
Supplement: Supplementary file 1 [file mmc1.docx]

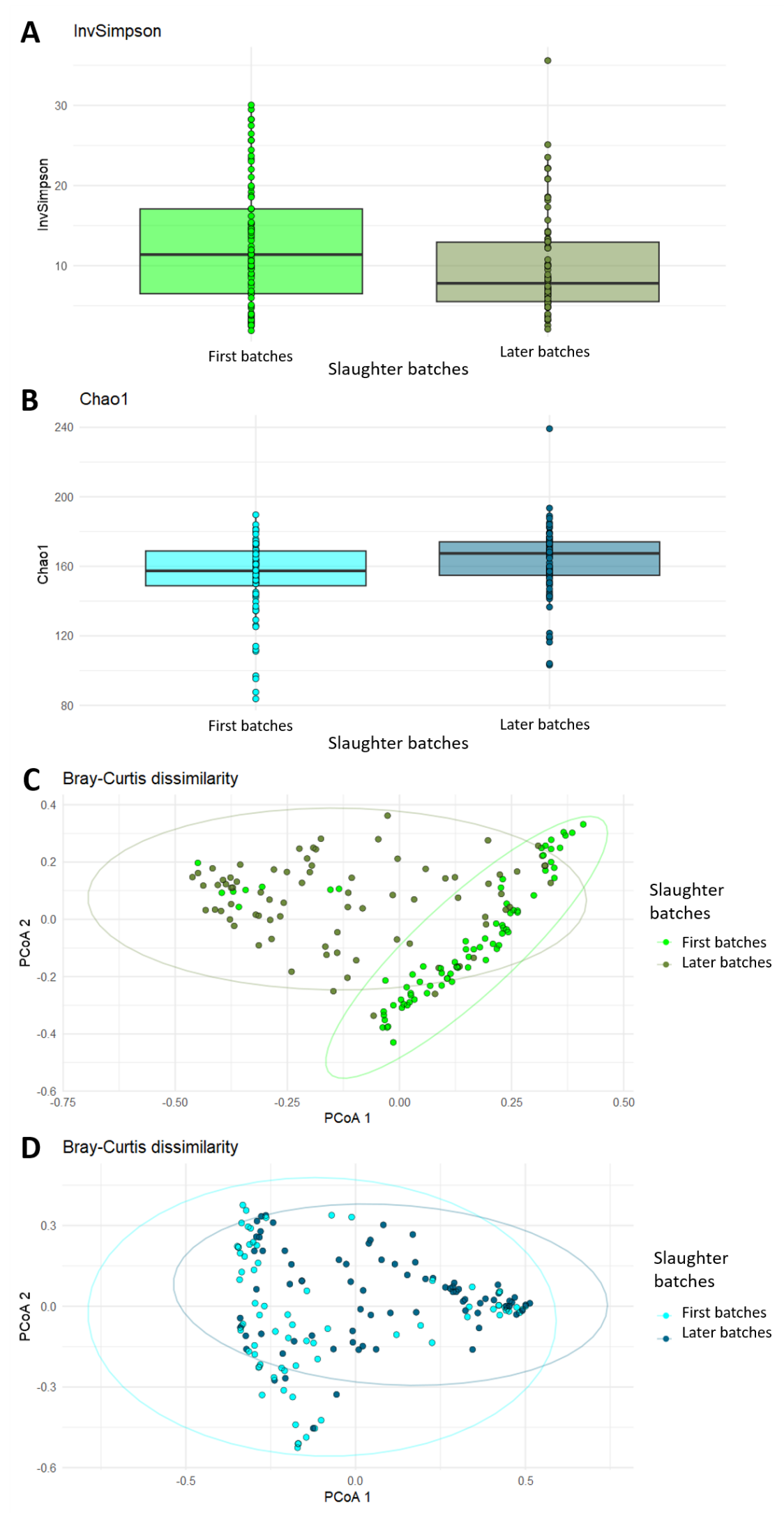


**Supplementary Figure 1. Alpha and beta diversity according to slaughter batches in conventional and free-range broiler carcasses. (A)** InvSimpson index (diversity) between first and later batches for conventional broilers. **(B)** Chao1 index (species richness) between first and later batches for free-range broilers. **(C)** Beta diversity between first and later batches for conventional broilers. **(D)** Beta diversity between first and later batches for free-range broilers.

**Supplementary Table 1. Metadata for each slaughter batch from which conventional and free-range (Label Rouge) carcasses were collected.** The batch N° refers to the slaughter batch of carcasses collected during our analysis (a total of 40 batches). It also includes the date and time when the poultry were slaughtered. Two different batches were collected on the same day (same date), but one corresponds to the first batch of poultry slaughtered during the day ("First"), and the other corresponds to the last or a later batch ("Later"). All broilers of the same type were slaughtered in the same specialized slaughterhouse (1 or 2), but the breeder could differ between broilers. Breeder and slaughterhouses remain anonymous and are named through different numbers. The “slaughter rate” correspond to the number of poultry slaughtered per hour for each batch.

| **Batch N°** | **Date** | **Slaughter time (am)** | **Slaughter batch** | **Broiler type** | **Slaughter rate** | **Breeder** | **Slaughterhouse** |
| --- | --- | --- | --- | --- | --- | --- | --- |
| 1 | 03/10/2022 | 02:20 | First | Conventional | 11300 | Breeder 1 | Slaughterhouse 1 |
| 2 | 03/10/2022 | 12:40 | Later | Conventional | 10800 | Breeder 2 | Slaughterhouse 1 |
| 3 | 06/10/2022 | 02:20 | First | Conventional | 11300 | Breeder 3 | Slaughterhouse 1 |
| 4 | 06/10/2022 | 12:10 | Later | Conventional | 10800 | Breeder 4 | Slaughterhouse 1 |
| 5 | 17/10/2022 | 02:20 | First | Conventional | 11300 | Breeder 5 | Slaughterhouse 1 |
| 6 | 17/10/2022 | 11:00 | Later | Conventional | 11300 | Breeder 6 | Slaughterhouse 1 |
| 7 | 03/11/2022 | 02:20 | First | Conventional | 11300 | Breeder 7 | Slaughterhouse 1 |
| 8 | 03/11/2022 | 11:25 | Later | Conventional | 11300 | Breeder 8 | Slaughterhouse 1 |
| 9 | 17/11/2022 | 02:20 | First | Conventional | 11300 | Breeder 9 | Slaughterhouse 1 |
| 10 | 17/11/2022 | 12:25 | Later | Conventional | 10800 | Breeder 10 | Slaughterhouse 1 |
| 11 | 28/11/2022 | 04:30 | First | Conventional | 11300 | Breeder 11 | Slaughterhouse 1 |
| 12 | 28/11/2022 | 09:50 | Later | Conventional | 10800 | Breeder 12 | Slaughterhouse 1 |
| 13 | 01/12/2022 | 07:15 | First | Conventional | 10800 | Breeder 13 | Slaughterhouse 1 |
| 14 | 01/12/2022 | 08:55 | Later | Conventional | 10800 | Breeder 14 | Slaughterhouse 1 |
| 15 | 12/12/2022 | 02:15 | First | Conventional | 11300 | Breeder 6 | Slaughterhouse 1 |
| 16 | 12/12/2022 | 12:00 | Later | Conventional | 11300 | Breeder 6 | Slaughterhouse 1 |
| 17 | 14/12/2022 | 02:20 | First | Conventional | 11300 | Breeder 15 | Slaughterhouse 1 |
| 18 | 14/12/2022 | 11:30 | Later | Conventional | 9500 | Breeder 16 | Slaughterhouse 1 |
| 19 | 05/01/2023 | 02:15 | First | Conventional | 10800 | Breeder 17 | Slaughterhouse 1 |
| 20 | 05/01/2023 | 07:30 | Later | Conventional | 9500 | Breeder 18 | Slaughterhouse 1 |
| 21 | 25/09/2023 | 0 :15 | First | Label Rouge | 7800 | Breeder 19 | Slaughterhouse 2 |
| 22 | 25/09/2023 | 3 :30 | Later | Label Rouge | 7800 | Breeder 20 | Slaughterhouse 2 |
| 23 | 28/09/2023 | 0 :15 | First | Label Rouge | 7800 | Breeder 21 | Slaughterhouse 2 |
| 24 | 28/09/2023 | 9 :40 | Later | Label Rouge | 7800 | Breeder 22 | Slaughterhouse 2 |
| 25 | 09/10/2023 | 0 :15 | First | Label Rouge | 7800 | Breeder 23 | Slaughterhouse 2 |
| 26 | 09/10/2023 | 4 :00 | Later | Label Rouge | 7800 | Breeder 24 | Slaughterhouse 2 |
| 27 | 12/10/2023 | 0 :15 | First | Label Rouge | 7800 | Breeder 25 | Slaughterhouse 2 |
| 28 | 12/10/2023 | 5 :10 | Later | Label Rouge | 7800 | Breeder 26 | Slaughterhouse 2 |
| 29 | 06/11/2023 | 1 :20 | First | Label Rouge | 7800 | Breeder 27 | Slaughterhouse 2 |
| 30 | 06/11/2023 | 3 :10 | Later | Label Rouge | 7800 | Breeder 28 | Slaughterhouse 2 |
| 31 | 23/11/2023 | 0 :15 | First | Label Rouge | 7800 | Breeder 29 | Slaughterhouse 2 |
| 32 | 23/11/2023 | 8 :20 | Later | Label Rouge | 7800 | Breeder 30 | Slaughterhouse 2 |
| 33 | 09/01/2024 | 1 :03 | First | Label Rouge | 7800 | Breeder 31 | Slaughterhouse 2 |
| 34 | 09/01/2024 | 3 :00 | Later | Label Rouge | 7800 | Breeder 32 | Slaughterhouse 2 |
| 35 | 17/01/2024 | 7 :38 | First | Label Rouge | 7800 | Breeder 33 | Slaughterhouse 2 |
| 36 | 17/01/2024 | 11 :09 | Later | Label Rouge | 7800 | Breeder 34 | Slaughterhouse 2 |
| 37 | 30/01/2024 | 10 :53 | First | Label Rouge | 7800 | Breeder 35 | Slaughterhouse 2 |
| 38 | 30/01/2024 | 11 :54 | Later | Label Rouge | 7800 | Breeder 36 | Slaughterhouse 2 |
| 39 | 06/02/2024 | 10 :38 | First | Label Rouge | 7800 | Breeder 37 | Slaughterhouse 2 |
| 40 | 06/02/2024 | 12 :51 | Later | Label Rouge | 7800 | Breeder 38 | Slaughterhouse 2 |

**Supplementary Table 2. Results of distribution fitting, including AIC, BIC and parameter estimates (with 95% confidence interval) for *Campylobacter* and TVC levels in conventional and free-range broiler carcasses.**

|  | Lognormal | Normal | Logistic | | Weibull | Gamma |
| --- | --- | --- | --- | --- | --- | --- |
| ***Campylobacter* –**  **Conventional carcasses** | AIC = 200.2  BIC = 205.7 | AIC = 193.2  BIC = 198.7 | | AIC = 192.7  BIC = 198.5 | AIC = 194.5  BIC = 200.1 | AIC = 194.2  BIC = 199.7 |
| ***Campylobacter* –**  **Free-range carcasses** | AIC = 361.4  BIC = 367.6 | AIC = 357.6  BIC = 363.8 | | AIC = 354.5  BIC = 360.6 | AIC = 353.6  BIC = 359.8 | AIC = 354.9  BIC = 361.1 |
| **TVC**  **–**  **Conventional carcasses** | AIC = 142.5  BIC = 148.7 | AIC = 156.0  BIC = 162.2 | | AIC = 142.6  BIC = 148.7 | AIC = 209.3  BIC = 215.4 | AIC = 146.6  BIC = 152.7 |
| **TVC**  **–**  **Free-range carcasses** | AIC = 361.4  BIC = 367.6 | AIC = 357.7  BIC = 363.8 | | AIC = 354.5  BIC = 360.6 | AIC = 353.7  BIC = 359.8 | AIC = 355  BIC = 361.1 |
|  |  |  | |  |  |  |

**Supplementary Table 3. List of the 50 discriminative genera distinguishing microbiota from conventional carcasses collected on the first or in the later slaughter batches.** The genera are mentioned along with their associated group (First batches *vs*. Later batches).

| **Genus** | **Group** |
| --- | --- |
| *Vogesella* | Later batches |
| *Bacillus* | Later batches |
| *Clostridium* sensu stricto 7 | Later batches |
| *Moraxella* | Later batches |
| *Caloramator* | Later batches |
| *Clostridium* sensu stricto 1 | Later batches |
| *Clostridium* sensu stricto 10 | Later batches |
| *Arcobacter* | First batches |
| *Anoxybacillus* | Later batches |
| *Tepidiphilus* | Later batches |
| *Acinetobacter* | First batches |
| *Simplicispira* | First batches |
| *Anaerococcus* | Later batches |
| *Romboutsia* | Later batches |
| *Luteimonas* | First batches |
| *Chryseobacterium* | First batches |
| *Brevundimonas* | First batches |
| *Gallibacterium* | Later batches |
| *Flavobacterium* | First batches |
| *Janthinobacterium* | First batches |
| *Pedobacter* | First batches |
| *Aneurinibacillus* | Later batches |
| *Pseudomonas* | First batches |
| *Schlegelella* | First batches |
| *Cloacibacterium* | First batches |
| *Enhydrobacter* | First batches |
| *Arthrobacter* | First batches |
| *Alistipes* | First batches |
| *Lactococcus* | First batches |
| *Bifidobacterium* | Later batches |
| *Tepidimonas* | First batches |
| *Vulcaniibacterium* | First batches |
| *Brochothrix* | First batches |
| *Clavibacter* | First batches |
| *Enterococcus* | Later batches |
| *Rikenellaceae* RC9 gut group | Later batches |
| *Staphylococcus* | First batches |
| *Aeromonas* | First batches |
| UCG-005 | Later batches |
| *Lactobacillus* | First batches |
| *Helicobacter* | Later batches |
| *Pseudarthrobacter* | First batches |
| *Psychrobacter* | First batches |
| *Ligilactobacillus* | Later batches |
| CHKCI001 | Later batches |
| [*Ruminococcus*] *gauvreauii* group | Later batches |
| *Christensenellaceae* R-7 group | Later batches |
| *Comamonas* | First batches |
| *Bacteroides* | Later batches |
| *Lysinibacillus* | First batches |
